# Evaluation of WNT Signaling Pathway Gene Variants *WNT7B* rs6519955, *SFRP4* rs17171229 and *RSPO2* rs611744 in Patients with Dupuytren’s Contracture

**DOI:** 10.3390/genes12091293

**Published:** 2021-08-24

**Authors:** Gediminas Samulėnas, Alina Smalinskienė, Rytis Rimdeika, Kęstutis Braziulis, Mantas Fomkinas, Rokas Paškevičius

**Affiliations:** 1 Department of Plastic and Reconstructive Surgery, Lithuanian University of Health Sciences, LT 50009 Kaunas, Lithuania; rytis.rimdeika@kaunoklinikos.lt (R.R.); kestutisbr@gmail.com (K.B.); mfomkinas@gmail.com (M.F.); 2 Laboratory of Molecular Cardiology, Institute of Cardiology, Lithuanian University of Health Sciences, LT 50103 Kaunas, Lithuania; 3 Institute of Biology Systems and Genetics Research, Lithuanian University of Health Sciences, LT 50103 Kaunas, Lithuania; 4 Faculty of Medicine, Lithuanian University of Health Sciences, LT 50009 Kaunas, Lithuania; rokaspaskevicius@gmail.com

**Keywords:** WNT signaling pathway, Dupuytren’s contracture, single-nucleotide polymorphism

## Abstract

Dupuytren’s contracture (DC) represents a chronic fibroproliferative pathology of the palmar aponeurosis, which leads to flexion contractures of finger joints and hand disability. In recent decades, the WNT signaling pathway has been revealed to play a significant role in the manifestation and pathogenesis of DC. Our study aimed to evaluate the associations between Dupuytren’s contracture and WNT-related single-nucleotide polymorphisms: Wnt Family Member 7B (*WNT7B)* rs6519955 (G/T), Secreted Frizzled Related Protein 4 (*SFRP4*) rs17171229 (C/T) and R-spondin 2 (*RSPO2)* rs611744 (A/G). We enrolled 216 patients (113 DC cases and 103 healthy controls), and DNA samples were extracted from the peripheral blood. Genotyping of *WNT7B* rs6519955, *SFRP4* rs17171229 and *RSPO2* rs611744 was performed using the Real-Time PCR System 7900HT from Applied Biosystems. *WNT7B* rs6519955 genotype TT carriers were found to possess a higher prevalence of DC (OR = 3.516; CI = 1.624–7.610; *p* = 0.001), whereas *RSPO2* rs611744 genotype GG appears to reduce the likelihood of the manifestation of DC nearly twofold (OR = 0.484, CI = 0.258–0.908, *p* = 0.024). In conclusion, SNPs *WNT7B* rs6519955 and *RSPO2* rs611744 are associated with the development of Dupuytren’s contracture: *WNT7B* rs6519955 TT genotype increases the chances by 3.5-fold, and *RSPO2* rs611744 genotype GG appears to attenuate the likelihood of the manifestation of DC nearly twofold. Findings of genotype distributions among DC patients and control groups suggest that *SFRP4* rs17171229 is not significantly associated with development of the disease.

## 1. Introduction

Dupuytren’s contracture (DC) represents a chronic fibroproliferative pathology of the palmar aponeurosis, which leads to flexion contractures of finger joints and hand disability. Genetic factors play a major role in the development of this condition; however, the exact cause remains unknown [1,2,3,4,5,6]. DC mainly affects middle-aged and elderly males and is mostly prevalent in Northern European populations, along with sporadic cases worldwide [7,8]. Recent decades have demonstrated great discoveries, narrowing the research range of DC towards distinct molecular mechanisms. The most important finding is the role of the WNT signaling pathway in the pathogenesis of Dupuytren’s contracture [2,4,9,10,11,12,13,14,15,16,17,18,19,20]. *WNT* genes encode proteins responsible for extracellular signaling. Alterations in this molecular transduction system are associated with fibrosis and a variety of diseases, including cancer [2,4,9,12,19]. Interestingly, the development of DC, due to its chronic proliferative nature, actually resembles an oncologic process—early Dupuytren’s contracture is histopathologically similar to fibrosarcoma [19]. The link between Dupuytren’s contracture and WNT signaling pathway was determined by locating abundant levels of β-catenin in the palmar fibro-aponeurotic tissue—the histological sequel of DC [11,12]. This multifunctional protein is the key factor of the WNT/β-catenin (canonical) pathway. Due to decreased degradation, β-catenin accumulates in the nucleus, binding to specific targets and activates prospective target genes [12]. SNPs from six loci (*WNT2* (rs4730775) (*p* = 3.0 × 10^−8^; OR, 0.83), *WNT4* (rs7524102) (*p* = 2.8 × 10^−9^; OR, 1.28), *SFRP4* (rs16879765) (*p* = 5.6 × 10^−39^; OR, 1.98), *SULF1* (rs2912522) (*p* = 2.0 × 10^−13^; OR, 0.72), *RSPO2* (rs611744) (*p* = 7.9 × 10^−15^; OR, 0.75) and *WNT7B* (rs6519955) (*p* = 3.2 × 10^−33^; OR, 1.54), harboring genes linked to the WNT signaling pathway, were discovered in a genome-wide association study by Dolmans et al., directly proving the association between Dupuytren’s contracture and this complex biosignaling system [4]. These results were later further endorsed and supplemented by Becker et al., providing even more WNT-related genes involved in the pathogenesis of DC [5].

We took the opportunity to evaluate the diversity of *WNT7B* rs6519955, *SFRP4* rs17171229 and *RSPO2* rs611744 between DC patients and healthy controls in a Lithuanian population. To the best of our knowledge, this is the frst genetic study of Dupuytren’s contracture in the Lithuanian population, who reside in a region with greatly increased genetic susceptibility to this illness [7,8]. 

## 2. Materials and Methods

This study included patients receiving day-case surgical treatment in the Department of Plastic and Reconstructive Surgery, Hospital of LUHS. A total of 216 patients accepted to participate and had their peripheral blood samples collected—there were 113 DC patients and 103 healthy controls. Detailed group demographics and characteristics are summarized in Table 1. The study group included patients with a clinical diagnosis of Dupuytren’s contracture. The control group comprised randomly selected patients who exhibited no clinical signs of DC or stenosing tenosynovitis, had no family history of DC, and had not been diagnosed or treated for this condition before [7,20]. Patients aged <30 years were not included in this study. Data on general medical status and underlying illnesses were acquired on examination and from medical records. All patients represented the same ethnic group of native Lithuanians.

### 2.1. Ethics Statement

Permissions to implement this study were granted by Kaunas Regional Biomedical Research Ethics Committee (No. BE-2-21, 2019-03-08) and the Department of Bioethics, LUHS (BEC-MF-63. 2017-10-23). All patients provided a written informed consent according to the Declaration of Helsinki. 

### 2.2. DNA Extraction and Genotyping

Genotyping of *WNT7B* rs6519955, *SFRP4* rs17171229 and *RSPO2* rs611744 was performed at the Molecular Cardiology Laboratory, LUHS. Blood samples for DNA extraction were harvested to EDTA tubes. DNA from peripheral blood leucocytes was obtained using a Thermo Fisher Scientific genomic DNA purification kit according to the manufacturer’s recommendations. Single-nucleotide polymorphisms were estimated by using an Applied Biosystems genotyping kits (*WNT7B* gene (rs6519955) C__11519407_1, *SFRP4* gene (rs17171229) C__34101042_20 and *RSPO2* gene (rs611744) C___1295706_20). A Real-Time PCR System 7900HT (Applied Biosystems, Foster City, CA, USA) was employed for SNP discovery. The cycling program was initiated by heating at 95 °C for 10 min, followed by 40 cycles (15 s at 95 °C and 1 min at 60 °C). Finally, allelic discrimination was performed using Applied Biosystems SDS 2.3 software.

### 2.3. Statistical Analysis

Statistical analysis was performed using the SPSS Statistics software (version 27.0). Data are provided as absolute numbers with percentages. Frequency scores of genotypes are expressed in percentages. The distributions of *WNT7B* rs6519955, *SFRP4* rs17171229 and *RSPO2* rs611744 between groups were analyzed using the chi-squared test. Binary logistic regression was used to evaluate the impact of the studied genotypes for the development of DC. Results were statistically significant when *p* was less than 0.05. 

## 3. Results

### 3.1. Distributions of WNT7B rs6519955, SFRP4 rs17171229 and RSPO2 rs611744 Genotypes between Patients with Dupuytren’s Contracture and Control Subjects

The distributions of *WNT7B* gene variant rs6519955, *SFRP4* gene variant rs17171229 and *RSPO2* gene variant rs611744 between patients with Dupuytren’s contracture and controls are provided in Table 2. Genotype frequencies did not deviate from the Hardy–Weinberg equilibrium (HWE) (*p* > 0.05). We found statistically significant differences in distributions of *WNT7B* rs6519955 and *RSPO2* rs611744 gene profiles between the DC group and healthy controls (Table 2).

Binary logistic regression analysis revealed that *WNT7B* rs6519955 genotype TT increased the chances of developing Dupuytren’s contracture by 3.5-fold (OR = 3.516; CI = 1.624–7.610; *p* = 0.001), whereas the *RSPO2* rs611744 genotype GG appeared to attenuate the likelihood of the manifestation of DC nearly twofold (OR = 0.484, CI = 0.258–0.908, *p* = 0.024) (Table 3).

### 3.2. WNT7B rs6519955, SFRP4 rs17171229 and RSPO2 rs611744 Genotypes and Positive Family History

The relationship between our examined genotypes and the incidence of DC cases among relatives of DC patients was evaluated. In the DC group, 34 out of 113 cases were hereditary DCs. We found borderline statistical significance between *SFRP4* rs17171229 gene variants and a family history of Dupuytren’s contracture. The other two examined SNPs (*WNT7B* rs6519955 and *RSPO2* rs611744) did not exhibit statistically significant differences in terms of heritability (Table 4).

### 3.3. Other Correlations

No statistically significant differences were found when evaluating *WNT7B* rs6519955, *SFRP4* rs17171229 and *RSPO2* rs611744 gene variants and daily physical labor, previously sustained hand injuries (traumatic injuries and surgeries, excluding surgical treatment of DC), DC stages, and the severity of DC diathesis (bilateral involvement, positive family history, <50 years of age on the onset of DC, male gender) [7]. Additionally, we did not observe any statistical significance when comparing the combined impact of genotype versions of both *WNT7B* rs6519955 and *SFRP4* rs17171229 and the occurrence of DC. However, we found a higher number of smokers in the DC group (Table 5). Patients smoking >1 cigarette/week were considered smokers. Non-smokers included patients who denied any usage of tobacco products in the past or had quit smoking at least 10 years previously.

## 4. Discussion

This is the first study evaluating genetic characteristics of Dupuytren’s contracture in a Lithuanian population. The Northern European region falls into the area of increased genetic susceptibility to DC [7,8]. Palmar fibromatous cords—the clinical hallmarks of Dupuytren’s contracture—suggest a variety of cellular and cytokine imbalances, most notably, the domination of III type collagen, reinless myofibroblast proliferation, high levels of β-catenin and decreased fibroblast apoptosis [2,3,17,19]. This has led to a proven connection between the WNT signaling pathway and DC. [2,4,9,10,11,12,13,14,15,16,17,18,19,20]. Moreover, fibrosis as a general process in human diseases is closely linked with aberrant activity of the canonical (β-catenin) WNT pathway [18]. The latter is also a major pathway of oncogenic biosignaling [21,22,23,24]. This explains why DC pathogenesis during early stages may histopathologically mimic an oncological process [19]. Gene expression studies have showed the upregulation of *WNT7B* and *SFRP4* genes in the diseased palmar fibromatous nodes, compared to the unaffected palmar fascia. Upregulation and close association with α-smooth muscle actin (α-SMA) and β-catenin-expressing cells makes *WNT7B* a likely pro-fibrotic agent for DC [17]. On the background of increased WNT activity, WNT proteins (including *WNT7B*) promote the impairment of β-catenin degradation. This leads to the accumulation of β-catenin inside the nucleus, binding to T cell factor (TCF) family and lymphoid enhancer-binding protein family (LEF) transcription factors, thus activating transcription. This step-by-step array of biochemical reactions is best described by Moon et al [12]. Our study showed that the *WNT7B* rs6519955 genotype TT increases the likelihood of DC by 3.5-fold (*p* = 0.001; odds ratio, 3.516). This finding is consistent with the data from the GWAS by Dolmans et al., where this SNP displayed the most significant associations with DC (*p* = 3.2 × 10^−33^; odds ratio, 1.54) [4]. 

Another SNP—*RSPO2* rs611744—also reached genome-wide significance in the same GWAS by Dolmans et al. (*p* = 7.9 × 10^−15^; odds ratio, 0.75), highlighting the *RSPO2* rs611744 genotype GG as a remissive factor towards DC [4]. Congruent data came from the study by Ng et al. of the *RSPO2* rs611744 genotype GG (*p* = 9.92 × 10^−16^, odds ratio, 0.794) [23]. We also found the *RSPO2* rs611744 genotype GG to act as an attenuating element in connection with DC (*p* = 0.024; odds ratio, 0.484). R-spondins represent a small group of extracellular ligands, separate from WNT proteins, which are involved in the upregulation of the canonical WNT system. R-spondins act by binding to LRP5/6 (low-density lipoprotein receptor-related protein 5/6) and frizzled receptors, thus promoting β-catenin signaling. Moreover, they have an antagonistic role against a WNT suppressor—Dickkopf protein (DKK)—by competing against it for binding to the same LRP5/6 [24].

In contrast, we did not observe a link between *SFRP4* rs17171229 and DC. SFRPs play an inhibitory role towards WNT signaling. The role of SFRPs in the pathogenesis of DC was proven by Dolmans et al., although they highlighted a different SNP. However, our chosen SNP (*SFRP4* rs17171229) did not exhibit any significant association with Dupuytren’s contracture. This finding is converse to the data published in other studies [4,5]. We believe that this is due to a limitation of our study—a small sample size.

We have coincidentally discovered higher rates of smoking among DC patients compared to controls (*p* = 0.021). The effect of cigarette smoking is linked to vascular occlusion and microangiopathy, leading to chronic ischemic state of the palmar fascia and harmful effects of free radicals [10]. However, the association between smoking and Dupuytren’s contracture is controversial [25,26,27].

Our findings in relation to *WNT7B* rs6519955 and *RSPO2* rs611744 were consistent with the recent observations by other authors [4,5]. The results from a newly examined population support existing evidence on the role of WNT signaling in the development of Dupuytren’s contracture. Major progress in this field could possibly provide therapeutic possibilities for an illness which currently has no etiological treatment options available [28,29,30,31]. However, SNPs discovered to date represent just a part of the whole mechanism of DC pathogenesis, leaving much to research further. 

## 5. Conclusions

Single-nucleotide polymorphisms *WNT7B* rs6519955 and *RSPO2* rs611744 are associated with the development of Dupuytren’s contracture: *WNT7B* rs6519955 genotype TT increases the chances by 3.5-fold and *RSPO2* rs611744 genotype GG appears to attenuate the likelihood of the manifestation of DC nearly twofold. Findings of genotype distributions among DC patients and control groups suggest that *SFRP4* rs17171229 is not significantly associated with the development of disease. 

## Figures and Tables

**Table 1:** Characteristics of study groups.

	Dupuytren’s Contracture	Control Group	*p*-Value
Gender:			*p* < 0.05 *
Male	97 (85.8%)	68 (66%)	
Female	16 (14.2%)	35 (34%)	
Total	113 (100%)	103 (100%)	
Age (years)			
Male	59.57 (SD 10.82)	59.22 (SD 14.26)	*p* > 0.05
Female	64.13 (SD 8.59)	57.6 (SD 12.66)	*p* < 0.05
Total	60.21 (SD 10.62)	58.67 (SD 13.69)	*p >* 0.05

SD, standard deviation; * Chi-squared comparison of patients’ gender between study groups.

**Table 2:** Distribution of *WNT7B* rs6519955, *SFRP4* rs17171229 and *RSPO2* rs611744 genotypes in DC and control groups.

SNP	Genotype	DC Group *n* = 113	Control Group *n* = 103	*p*-Value
*WNT7B* rs6519955	GG	24 (21.2%)	32 (31.1%)	***p* = 0.003**
	GT	58 (51.3%)	61 (59.2%)	
	TT	31 (27.4%)	10 (9.7%)	
	GG + GT	82 (72.6%)	93 (90.3%)	***p* = 0.001**
	TT	31 (27.4%)	10 (9.7%)	
*SFRP4* rs17171229	CC	7 (6.2%)	5 (4.9%)	*p* > 0.05
	CT	41 (36.3%)	23 (22.3%)	
	TT	65 (57.5%)	75 (72.8%)	
	CT + TT	106 (93.8%)	98 (95.1%)	*p* > 0.05
	CC	7 (6.2%)	5 (4.9%)	
*RSPO2* rs611744	AA	39 (34.5%)	20 (19.4%)	***p* = 0.015**
	AG	53 (46.9%)	50 (48.5%)	
	GG	21 (18.6%)	33 (32%)	
	AA + AG	92 (81.4%)	70 (68%)	***p* = 0.023**
	GG	21 (18.6%)	33 (32%)	

DC, Dupuytren’s contracture; SNP, single-nucleotide polymorphism; statistically significant results in **bold** (when *p* < 0.05).

**Table 3:** The impact of *WNT7B* rs6519955, *SFRP4* rs17171229 and *RSPO2* rs611744 on the development of DC.

SNP	Genotype	OR (95% CI)	*p*-Value
*WNT7B* rs6519955	TT vs. GT	4.133 (1.701–10.043)	**0.002**
	TT vs. GG	3.260 (1.467–7.244)	**0.004**
	TT vs. GT + GG	3.516 (1.624–7.610)	**0.001**
*SFRP4* rs17171229	CC vs. CT	1.615 (0.489–5.335)	0.431
	CC vs. TT	0.785 (0.224–2.758)	0.706
	CC vs. CT + TT	1.294 (0.398–4.212)	0.668
*RSPO2* rs611744	GG vs. AG	0.326 (0.151–0.703)	**0.004**
	GG vs. AA	0.600 (0.307–1.173)	**0.135**
	GG vs. AG + AA	0.484 (0.258–0.908)	**0.024**

SNP, single-nucleotide polymorphism; OR, odds ratio; statistically significant results in **bold** (when *p* < 0.05).

**Table 4:** Distribution of *WNT7B* rs6519955, *SFRP4* rs17171229 and *RSPO2* rs611744 genotypes in the DC group by heritability.

SNP	Genotype	Positive Family History	Negative Family History	*p*-Value
*WNT7B* rs6519955	GG	5	19	*p* > 0.05
	GT	19	39	
	TT	10	21	
	Total	34	79	
*SFRP4* rs17171229	CC	2	5	***p* = 0.05**
	CT	18	23	
	TT	14	51	
	Total	34	79	
*RSPO2* rs611744	AA	13	26	*p* > 0.05
	AG	12	41	
	GG	9	12	
	Total	34	79	

SNP, single-nucleotide polymorphism; statistically significant results in **bold** (when *p* <= 0.05).

**Table 5:** Distribution of smokers and non-smokers between patient groups.

	DC Group	Control Group	
Smokers	39	21	***p* = 0.021**
Non-smokers	74	82	

Statistically significant results in **bold** (when *p* <= 0.05).

## Data Availability

Data supporting the reported results are available directly from the main author upon request.
